# Internalized Stigma and Help-Seeking Across Problems and Targets

**DOI:** 10.1007/s11414-025-09981-z

**Published:** 2025-11-11

**Authors:** A. Grace Kelly, Natasha A. Tonge

**Affiliations:** Department of Psychology, George Mason University, 4400 University Drive, Fairfax, VA 22030 USA

## Abstract

Mental health difficulties have been on the rise in recent years. Self-disclosure of mental health symptoms is related to a range of positive outcomes, and seeking help for mental health can be life-saving. Help-seeking for one’s mental health can be impacted by a variety of factors, such as the help-seeking target (e.g., friend, family member, or professional source) and the presenting problem (e.g., a personal emotional problem vs suicidal ideation). Internalized stigma of mental illness can impede help-seeking; however, it is not known if internalized stigma impedes (1) only certain types of problems or (2) help-seeking only from certain targets. It was hypothesized that the relationship between internalized stigma and willingness to seek help would vary based on (1) help-seeking target and (2) whether someone is seeking help for a personal emotional problem or suicidal thinking. Utilizing a sample of undergraduate students and a sample recruited through Prolific of adults with a history of help-seeking for mental health, bifactor models were run with stigma included as a manifest variable predicting willingness to seek help for suicidal ideation and personal emotional problems (each included as general factors) from three types of targets (friends/intimate partners, family members, and professional sources, each included as specific factors). Results indicate that internalized stigma negatively predicts willingness to seek help for suicidal thinking across targets and negatively predicts willingness to seek help for personal/emotional problems from friends/intimate partners and family members, but not from professional targets. These findings demonstrate that the relationship between internalized stigma and willingness to seek help is not uniform across help-seeking targets and presenting problems.

Mental health difficulties have been on the rise in recent years. In 2021, nearly half of Americans surveyed experienced significant symptoms of anxiety and depression.^1,2^ Even more concerning, suicide is a leading cause of death in the USA, with suicide rates having increased by 36% between 2000 and 2021.^3^ Anxiety, depression, and suicidal ideation continue to carry significant societal stigma, and people may be reluctant to seek help for these conditions.^4–6^ Furthermore, when people experience societal stigma, they may go on to internalize those stigmatizing beliefs.^7^ It is known that internalized stigma can lead to both exacerbated psychological distress and decreased likelihood of help-seeking, but it is not known how internalized stigma may impact willingness to seek help differently across distinct help-seeking targets and presenting problems.^8–10^

Seeking help for suicidal ideation can be lifesaving: disclosure and subsequent help-seeking are necessary precursors to receiving treatment for those who need it. Further, self-disclosure of mental health symptoms is related to a range of positive outcomes, such as enhanced social support and improved quality of life.^4–6^ Failure to seek needed help for one’s mental health can lead to exacerbated psychological distress, diminished quality of life, and personal and societal financial costs.^11–14^ Despite the array of benefits of disclosing and the costs of failing to do so, many people do not disclose their mental health struggles or seek help for them.^15,16^ Because help-seeking is a crucial link in the path to potentially life-saving treatment, it is important to understand what factors influence the likelihood of willingness to seek help.

Accounting for the help-seeking target is an important factor when assessing predictors of help-seeking. People seek help for their mental health struggles from a variety of sources, such as friends, family, mental health professionals, and primary care providers, and people may seek help from distinct targets in different circumstances.^17–19^ For example, if someone turns to a friend for help with their mental health, they may hope to receive emotional support, whereas if someone seeks help from a mental health provider, they likely do so with the goal of engaging in treatment.^20^ However, research examining whether factors associated with willingness to seek help vary across different help-seeking targets is surprisingly sparse.

Internalized stigma, which occurs when people incorporate negative stereotypes about mental illness into their own self-image, is a well-documented correlate of self-disclosure and help-seeking.^9,15,21–24^ Research has not yet assessed whether the relationship between internalized stigma and willingness to seek help varies based on help-seeking target. While research indicates that self-stigma of seeking help inhibits help-seeking from both formal and informal targets broadly, self-stigma of seeking help and internalized stigma of mental illness are separate constructs that can function differently from each other.^16,25^ Because prior research indicates that internalized stigma of mental illness is a crucial predictor of help-seeking, further research is needed to demonstrate how this construct may predict willingness to seek help across various targets.^9,26^

Past research indicates that the nature of the presenting psychological distress also influences the likelihood of help-seeking.^27^ One key mental health symptom that may differ from others in this regard is suicidal ideation. Disclosing suicidal ideation can lead to more extreme consequences such as involuntary hospitalization, increased stigma, and fear-based negative reactions from others compared to disclosing less severe symptoms of depression, anxiety, or general emotional distress.^28–30^ Relatedly, qualitative research indicates that university students may avoid disclosing thoughts of suicide or self-harm more so than other concerns such as depression or anxiety symptoms, due to fear of stigmatizing reactions from others.^31^ Because the decision to seek help for suicidality is distinct from the decision to seek help for other mental health concerns, it is possible that the relationship between internalized stigma and willingness to seek help varies based on whether someone is seeking help for suicidal ideation or another mental health concern. On one hand, someone high in internalized stigma may be less likely to seek help for suicidal ideation because of the increased stigma attached to suicide. On the other hand, the heightened urgency of suicidal ideation may weaken the relationship between internalized stigma and willingness to seek help, as the need for immediate help may serve to “override” the effect of stigma. However, to the authors’ knowledge, past research has not investigated this topic, and it is not yet known whether the relationship between internalized stigma and willingness to seek help varies based on the nature of the presenting problem.

In summary, not only is it possible that the relationship between internalized stigma and willingness to seek help differs depending on the nature of the presenting problem (e.g., general distress vs suicidal ideation) but also the relationship between internalized stigma and willingness to seek help may vary depending on the help-seeking target. What is more, the relationship between internalized stigma and willingness to seek help for each target may differ depending on the presenting problem, as people may seek help from different sources based on the presenting problem. People may feel more comfortable discussing different concerns with different sources, and stigma may play a different role depending on both the presenting concern and help-seeking target. Internalized stigma is a key predictor of mental health help-seeking, but it is not known whether the relationship between internalized stigma and help-seeking is uniform across help-seeking targets and presenting problems. Thus, in the current study, the authors sought to investigate whether the relationship between internalized stigma and willingness to seek help differed based on the help-seeking target and the nature of the presenting problem (i.e., a general personal/emotional problem vs suicidal thinking).

The authors first conducted a pilot project to investigate the factor structure of the General Help-Seeking Questionnaire.^32^ The GHSQ is a widely used measure of help-seeking. However, despite the prevalence of the GHSQ in the help-seeking literature, research exploring its psychometric properties is sparse, and it was unknown which help-seeking targets tended to group together.

Based on results from the pilot study (described in detail in the “Method” and “Results” sections of this manuscript), the authors hypothesized that internalized stigma would show different relationships with willingness to seek help based on whether the individual was seeking help from (1) family, (2) friends and intimate partners, or (3) professional sources, and whether the individual was seeking help for a general personal/emotional problem or for suicidal thinking (see Fig. 1).

**Figure 1:**
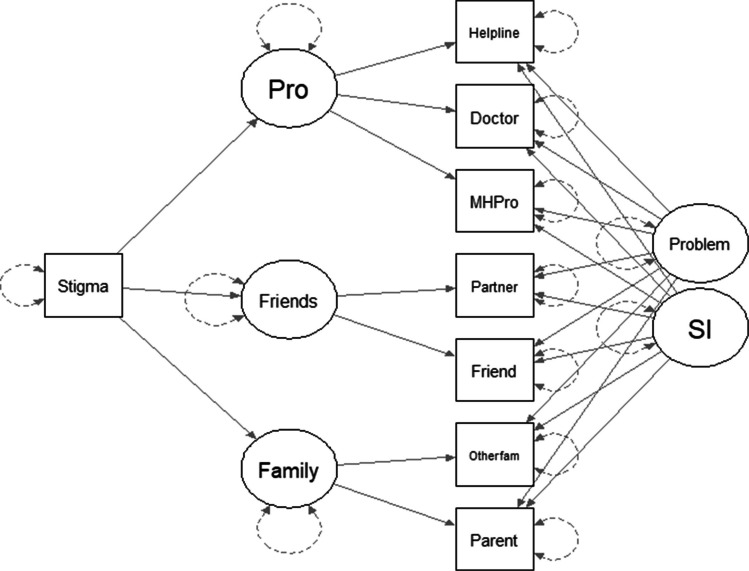
Hypothesized Model of Internalized Stigma and Help-Seeking This figure depicts our hypothesized bifactor model, with internalized stigma (“Stigma”) predicting help-seeking for suicidal thinking (“SI”) and personal/emotional problems (“Problem”) from professionals (“Pro”), friends/intimate partners (“Friends”), and family members (“Family”)

The authors first examined these relationships in a sample of undergraduates (Sample 1), and then sought to replicate findings from Sample 1 in a sample of participants with a history of help-seeking for mental health recruited from Prolific (Sample 2). Including a secondary sample of adults with a history of help-seeking allowed the authors to determine whether results from the undergraduate sample (which was not selected based on a history of help-seeking) would extend to individuals with a direct history of navigating mental health services. Undergraduates present with unique developmental and contextual features relative to mental health concerns and health service utilization.^33–37^ For example, while undergraduates may have more straightforward pathways to mental health services through the infrastructure provided by universities, some research indicates that undergraduates have lower rates of help-seeking for their mental health.^37^ Moreover, late adolescence and early adulthood represent a critical point in development, with many college students experiencing symptoms of mental illness for the first time.^38–40^ In contrast, the secondary sample included adults living within various environments, diverse life stages, and direct experience seeking help for mental health. A multi-sample design allowed for examination of relationships between internalized stigma and willingness to seek help across populations differing in life stage, environment, and experience. Testing hypotheses across multiple samples aligns with methodological recommendations for enhancing reproducibility and external validity in psychological research.^41,42^

## Method

### Participants

#### Pilot study

The authors recruited 144 undergraduate students at a large, diverse, public university on the East Coast of the USA. Participants were recruited through the university’s Sona systems subject pool and were compensated with course credit. All participants were ages 18 or older and spoke sufficient English to complete the study surveys. After filtering the data based on participants spending an average of at least 2 seconds on each survey item (as recommended by Bowling et al.^43^), analyses were conducted on a sample of 135. Demographics of the 135 participants included in analyses are presented in Table 1.

**Table 1:** Demographics

	Pilot sample		Sample 1	Sample 2
*n*		135	615	530
Age (mean (SD))		-	20.24 (3.38)	39.72 (12.82)
ISMI-29 (mean (SD))		-	38.91 (13.07)	47.44 (14.02)
GHSQ – personal/emotional				
Friends/intimate partner		10.11 (3.13)	10.10 (2.82)	10.01 (3.06)
Family		7.61 (3.5)	7.50 (3.25)	7.31 (3.50)
Professional		9.41 (4.49)	9.29 (4.26)	12.90 (4.11)
GHSQ – suicidal thinking				
Friends/intimate partner		9.08 (3.65)	9.27 (3.59)	8.88 (3.78)
Family		7.08 (4.17)	6.81 (3.82)	6.13 (3.80)
Professional		11.02 (5.66)	10.83 (5.23)	13.55 (5.15)
Race				
White		46 (34.1%)	244 (39.7%)	393 (74.2%)
Black		13 (9.6%)	74 (12.0%)	40 (7.5%)
Hispanic		31 (23.0%)	87 (14.1%)	35 (6.6%)
Asian		34 (35.2%)	151 (24.6%)	24 (4.5%)
American Indian		0 (0%)	1 (0.2%)	5 (0.9%)
Pacific Islander		1 (0.7%)	2 (0.3%)	3 (0.6%)
Multiracial		9 (6.7%)	51 (8.3%)	30 (5.7%)
Not answered		1 (0.7%)	5 (0.8%)	0 (0%)
Bicultural identity				
Not bicultural		62 (45.9%)	328 (53.3%)	413 (77.9%)
Bicultural		72 (53.3%)	285 (46/3%)	117 (22.1%)
Not answered		1 (0.7%)	2 (0.3%)	0 (0%)
Language at home				
English only		66 (48.9%)	323 (52.5%)	463 (87.4%)
Additional language		68 (50.4%)	291 (47.3%)	67 (12.6%)
Not answered		1 (0.7%)	1 (0.2%)	0 (0%)
Gender				
Cis-woman		89 (65.9%)	396 (64.4%)	339 (64.0%)
Cis-man		41 (30.4%)	159 (25.9%)	137 (25.8%)
Trans woman		0 (0%)	5 (0.8%)	4 (0.8%)
Trans man		0 (0%)	7 (1.1%)	14 (2.6%)
Non-binary		3 (2.2%)	29 (4.7%)	30 (5.7%)
Self-identify		0 (0%)	11 (1.8%)	2 (0.4%)
Prefer not to respond		1 (0.7%)	8 (1.3%)	4 (0.8%)
Sexual orientation				
Straight		99 (73.3%)	408 (66.3%)	357 (67.4%)
Bisexual		18 (13.3%)	100 (16.3%)	97 (18.3%)
Gay		1 (0.7%)	10 (1.6%)	15 (2.8%)
Lesbian		1 (0.7%)	21 (3.4%)	14 (2.6%)
Queer		4 (3.0%)	19 (3.1%)	19 (3.6%)
Questioning		6 (4.4%)	18 (2.9%)	5 (0.9%)
Self-identify		1 (0.7%)	22 (3.6%)	21 (4.0%)
Prefer not to respond		4 (3.0%)	17 (2.8%)	2 (0.4%)
Income				
Less than $10,000		-	34 (5.6%)	45 (8.5%)
$20,000–50,000		-	112 (18.4%)	158 (29.8%)
$50,000–100,000		-	195 (32.0%)	167 (31.5%)
$100,000–150,000		-	128 (21.0%)	78 (14.7%)
$ > 150,000		-	98 (16.1%)	48 (9.1%)
Not answered		-	42 (6.9%)	34 (6.4%)

### Sample 1

Sample 1 consisted of 651 undergraduate students at a large, diverse, public university on the East Coast of the USA, again recruited through the university subject pool and compensated with course credit. Inclusion criteria remained the same for Sample 1. Four data quality checks were introduced at various points throughout the survey. These items prompted participants to pay attention and answer questions honestly at the start of the survey, checked attention at two points during the survey, and assessed participants’ own opinions about the quality of their data at the end of the survey. Participants were awarded credit regardless of their answers to these questions, but only participants who passed three out of four data quality checks were included in analyses. After filtering the data based on passing three out of four data quality checks, spending a total amount of time on study surveys equivalent to an average of 2 seconds per item, and completing the study within 24 h (participants were permitted to pause the survey and return to it later), analyses were conducted on a final sample of 615; 59.5% of the sample reported having sought treatment for their mental health before. Demographics of the participants included in analyses are presented in Table 1.

### Sample 2

Sample 2 participants consisted of 534 adults with a reported history of help-seeking for mental health recruited from the online research recruitment platform Prolific. Participants were eligible if they reported a history of diagnosed or suspected mental health conditions, reported use of psychotropic medication, and a history of treatment seeking for mental health (as indicated through Prolific’s pre-set filters). All Sample 2 participants had a Prolific study approval rate between 95 and 100%. Participants were ages 18 or older and spoke sufficient English to complete study measures. As with Sample 1, participants completed 4 data quality checks. After filtering based on the same criteria used with Sample 1, the final sample for analysis was 530. 97.9% of the sample reported having sought treatment for their mental health before. Demographics of the participants included in analyses are presented in Table 1.

### Measures

Participants completed a demographics questionnaire and several other self-report questionnaires not used in the current study. Willingness to seek help for both personal/emotional problems and suicidal thinking was measured through the General Help-Seeking Questionnaire (GHSQ).^32^ The GHSQ assesses individuals’ likelihood of seeking help from a variety of sources (e.g., friend, intimate partner, parent, primary care physician) on a five-point Likert scale by asking, “If you were [‘having a personal or emotional problem’ or ‘experiencing suicidal thoughts’] how likely is it that you would seek help from the following people?.”

Internalized stigma of mental illness was assessed in Samples 1 and 2 using the Internalized Stigma of Mental Illness scale (ISMI).^44^ The ISMI is a 29-item measure that can be separated into five subscales (Alienation, Stereotype Endorsement, Discrimination Experience, Social Withdrawal, and Stigma Resistance) or assessed as a total score reflecting overall levels of internalized stigma. For analyses in this study, internalized stigma was computed as a total score, excluding the items comprising the Stigma Resistance subscale due to low reliability (Sample 1 *ɑ* = 0.43; Sample 2 *ɑ*= 0.35). This is consistent with practices from other studies utilizing this measure.^45^ Excluding the Stigma Resistance subscale, the ISMI total demonstrated strong reliability (Sample 1 *ɑ* = 0.93; Sample 2 *ɑ* = 0.90).

### Procedure

All procedures were approved by the university Institutional Review Board (IRB#s 2147546–3 and 1974781–3), and all three participant samples completed informed consent. Participants completed demographic measures, and study surveys through Qualtrics. Due to experimenter error, the pilot sample did not complete demographic information on their age and level of income. After completing the study, all participants were provided with a debriefing form summarizing key information about the study as well as contact information for mental health resources: for the pilot sample and Sample 1, these resources were local to the college, and for Sample 2, the resources provided were available nationally. Participants in the pilot sample and Sample 1 completed the study for course credit in an undergraduate psychology course. Participants in Sample 2 received compensation at a rate of $9 per hour for participating.

### Data analytic plan

Using the pilot sample, the authors conducted exploratory factor analyses of the GHSQ – Suicidal Thinking and GHSQ – Personal/Emotional Problem separately. The authors identified factors in the pilot sample for use with bifactor models with Samples 2 and 3.^46,47^ The bifactor model approach allowed examination of shared variance of all help-seeking items and facilitated parsing out the unique variance of willingness to seek help for a personal emotional problem or suicidal thinking and the unique variance of specific factors (e.g., factors identified in the EFA step). Examining the significance of paths between internalized stigma and willingness to seek help from each target allowed determination of relationships between stigma and willingness to seek help for each target. Missing data for all analyses were handled using listwise deletion. All analyses were conducted using R with the *lavaan*package.^65^^,66^

## Results

### Exploratory factor analysis with pilot sample

Factor loadings are presented in Tables 2 and 3. For personal/emotional problems, a three-factor structure emerged, with willingness to seek help from doctor/general practitioner, mental health professional, phone help-lines, and religious leaders loading onto one factor; parents and other family members loading onto another factor; and friends and intimate partners loading onto a third factor. For suicidal thinking, two potential factor structures emerged: a three-factor structure mirroring that of GHSQ – Personal/Emotional Problem (friends/intimate partners, professional sources, and family members) and a two-factor structure with doctor/general practitioner, mental health professional, phone help-lines, and religious leaders loading onto one factor and parents, other family members, friends, and intimate partners loading onto another. In order to keep consistent analyses across the two versions of the GHSQ and to allow for analysis of potential differences in the relationship between willingness to seek help for friends/intimate partners and family members, primary study hypotheses assumed a three-factor structure of both versions of the GHSQ: Professionals (Doctor/GP, Mental Health Professional, and Help-Lines), Family (Parents and Other Relative/Family Member), and Friends/Intimate Partners. Religious leaders were dropped from the professional factor in analyses for the primary study, as this item showed the weakest loading onto this factor in both the suicidal thinking and personal/emotional problem EFAs, because religious affiliation was not assessed in this study, and because religious leaders are theoretically distinct from the other items in this factor (i.e., secular professional sources).

**Table 2:** Pattern factor loadings for exploratory factor analysis of a GHSQ – personal/emotional problem

		Factor		
Item no	Items	1	2	3
1	Intimate partner (e.g., girlfriend, boyfriend, husband, wife, de’ facto)	**-**	-	**.60**
2	Friend (not related to you)	-	-	**.73**
3	Parent	-	**1.07**	**-**
4	Other relative/family member	-	**.46**	**-**
5	Mental health professional (e.g., psychologist, social worker, counsellor)	**.73**	-	**-**
6	Phone helpline (e.g., Lifeline)	**.73**	**-**	**-**
7	Doctor/GP	**.86**	-	-
8	Minister or religious leader (e.g., Priest, Rabbi, Chaplain)	.36	-	-
9	I would not seek help from anyone	**-**	−.38	−.33
	% of variance	**22**	17	11

Factor loadings are in boldface. Extraction method: maximum likelihood. Rotation method: promax rotation

**Table 3:** Pattern factor loadings for exploratory factor analysis of a GHSQ – suicidal thinking

		Factor		
Item no	Items	1	2	3
1	Intimate partner (e.g., girlfriend, boyfriend, husband, wife, de facto)	**-**	-	**.44**
2	Friend (not related to you)	-	-	**.31**
3	Parent	-	**.58**	**-**
4	Other relative/family member	-	**.95**	**-**
5	Mental health professional (e.g., psychologist, social worker, counsellor)	**.93**	−.30	.43
6	Phone helpline (e.g., Lifeline)	**.68**	**-**	**-**
7	Doctor/GP	**.75**	-	-
8	Minister or religious leader (e.g., Priest, Rabbi, Chaplain)	.**41**	-	-
9	I would not seek help from anyone	**-**	-	−.62
	% of variance	**22**	16	11

Factor loadings are in boldface. Extraction method: maximum likelihood. Rotation method: promax rotation

### Effects of stigma on general willingness to seek help in sample 1

First, the authors ran a model with specific factors for willingness to seek help from family members, friends/intimate partners, and professional sources and general factors for willingness to seek help for suicidal thinking and personal/emotional problems. However, this model did not converge. Therefore, the authors next ran separate models for willingness to seek help items related to personal/emotional problems and suicidal ideation.

#### Effects of stigma on willingness to seek help for personal/emotional problems in Sample 1

The authors first ran a model with willingness to seek help from family members, friends/intimate partners, and professional sources included as general factors. This model resulted in adequate fit (CFI = 0.986, TLI = 0.957, RMSEA = 0.049, SRMR = 0.023). Next, the authors added in internalized stigma as a manifest variable, with pathways to each disclosure target. This model resulted in good fit (CFI = 0.981, TLI = 0.951, RMSEA = 0.046, SRMR = 0.025). Stigma significantly negatively predicted willingness to seek help from family (*b* = −0.03, *p* < 0.001) and friends (*b* = −0.02, *p* = 0.002), but did not significantly predict willingness to seek help from professional sources (*b* = 0.010, *p* = 0.08).

#### Effects of stigma on willingness to seek help for suicidal thinking in sample 1

The authors followed the same approach for willingness to seek help for suicidal thinking as they did for personal/emotional problems. The initial model (with willingness to seek help from family members, friends/intimate partners, and professional sources included as general factors) demonstrated good fit (CFI = 0.987, TLI = 0.962, RMSEA = 0.061, SRMR = 0.020). Adding internalized stigma as a manifest variable resulted in good fit (CFI = 0.983, TLI = 0.958, RMSEA = 0.056, SRMR = 0.023). Stigma significantly negatively predicted willingness to seek help from family (*b* = −0.03, *p* < 0.001), friends (*b* = −0.02, *p* = 0.001), and professional sources (*b* = −0.02, *p* = 0.01).

In summary, stigma negatively predicted the likelihood of seeking help from family and friends/intimate partners, but not from professionals for personal/emotional problems. For suicidal thinking, on the other hand, stigma seems to impair willingness to seek help for all targets. Therefore, the hypotheses that the relationship between internalized stigma and willingness to seek help would vary based on willingness to seek help target and presenting problem were supported.

#### Effects of internalized stigma on general willingness to seek help in sample 2

As with the undergraduate sample (Sample 1), the authors first ran a model with specific factors for willingness to seek help from family members, friends/intimate partners, and professional sources, general factors for willingness to seek help for suicidal ideation and personal/emotional problems. This model converged but did not demonstrate a strong fit (CFI = 0.774, TLI = 0.611, RMSEA = 0.170, SRMR = 0.038). Therefore, the authors did not proceed with adding internalized stigma as a manifest variable, and instead first separated the model into two models—one with willingness to seek help for a personal/emotional problem, and one for willingness to seek help for suicidal thinking.

#### Effects of internalized stigma on willingness to seek help for personal/emotional problems in sample 2

A model with willingness to seek help from family members, friends/intimate partners, and professional sources included as general factors resulted in adequate fit (CFI = 0.928, TLI = 0.785, RMSEA = 0.092, SRMR = 0.038). Adding internalized stigma as a manifest variable with pathways to each disclosure target resulted in adequate fit (CFI = 0.911, TLI = 0.774, RMSEA = 0.087, SRMR = 0.042). Stigma significantly negatively predicted willingness to seek help from family (*b* = −0.02, *p* < 0.01) and friends (*b* = −0.02, *p* < 0.001), but did not significantly predict willingness to seek help from professional sources (*b* = 0.003, *p* = 0.43).

#### Effects of internalized stigma on willingness to seek help for suicidal thinking in sample 2

A model with willingness to seek help from family members, friends/intimate partners, and professional sources included as general factors resulted in good fit (CFI = 0.971, TLI = 0.912, RMSEA = 0.085, SRMR = 0.026). Adding in internalized stigma as a manifest variable resulted in good fit (CFI = 0.966, TLI = 0.914, RMSEA = 0.074, SRMR = 0.028). Stigma significantly negatively predicted willingness to seek help from professional sources (*b* = −0.02, *p* < 0.001) and friends (*b* = −0.03, *p* < 0.001), and marginally predicted willingness to seek help from family (*b* = −0.013, *p* = 0.06). In summary, results in Sample 1 mirrored those of Sample 2: stigma negatively predicted the likelihood of seeking help from family and friends/intimate partners, but not from professionals for personal/emotional problems. For suicidal thinking, on the other hand, stigma seemed to impair willingness to seek help for all targets.

## Discussion

This study examined relationships of internalized stigma and willingness to seek help across two presenting problems (i.e., personal/emotional problem and suicidal thinking) and three types of help-seeking targets (i.e., friends/intimate partners, family members, and professional sources). Results were examined from an undergraduate sample including individuals with and without a history of help-seeking for mental health and a Prolific sample of individuals with a history of help-seeking for their mental health.

The results of this study indicate that the relationship between internalized stigma and willingness to seek help is not uniform across help-seeking targets and presenting problems. For both Sample 1 (undergraduate) and Sample 2 (Prolific), stigma negatively predicted the likelihood of seeking help from family and friends/intimate partners, but not from professionals for personal/emotional problems. For suicidal thinking, on the other hand, stigma seems to impair willingness to seek help for all targets. While it is not possible to make definitive claims as to the reasons behind the apparent differences in relationships between stigma and willingness to seek help, the authors explore potential factors driving these discrepancies based on the extant literature.

The finding that internalized stigma does not impair willingness to seek help from professional sources for personal/emotional problems could be surprising given the existing help-seeking literature. Prior research indicates that people may tend to seek help from informal targets more often than formal targets, which could indicate that stigma is particularly salient in the context of seeking professional support.^6,16^ However, prior research has not considered how the presenting problem and the help-seeking target can work together to impact the relationship between internalized stigma and willingness to seek help. This study’s results expand on the current understanding of the relationship between internalized stigma and willingness to seek help from professional sources by demonstrating that this relationship may be influenced by the nature of the problem for which the individual is seeking support.

Prior research indicates that the anticipated outcome of help-seeking is a crucial factor influencing one’s decision to seek help.^48^ It is likely that the anticipated outcome of help-seeking for different targets varies based on internalized stigma. One potential negative outcome that may be salient for those high in internalized stigma is experiencing stigmatizing attitudes from those to whom they self-disclose. Internalized stigma is related to anticipated stigma, which occurs when an individual with a stigmatized identity expects to experience stigma from others.^49–51^ If someone is high in internalized stigma, then they may be more likely to expect that self-disclosure of a personal emotional problem may result in a friend or family member adopting stigmatizing attitudes towards them, which may outweigh the possible outcome of obtaining social support. In other words, someone high in internalized stigma may expect the primary outcome of their help-seeking from a friend or family member to be negative. However, the primary outcome of seeking help from a professional target may still be seen as positive. In a qualitative study of young people with high symptoms of depression, participants noted that disclosure of symptoms both opened them up to experiencing increased stigma and was a pathway to help.^52^ Even if someone is high in internalized stigma, they may view a professional target as the “correct” choice for help-seeking and believe that the primary outcome of receiving professional support outweighs the possible outcome of negative perception or stigma from the provider.

The negative perception of those outside of one’s social network (i.e., professional sources) may simply bear less weight than the negative perception of those within one’s own social network. The “need to belong,” or the desire to feel connected to others within one’s social circle, is considered a fundamental motivator of human behavior.^53^ It is possible that disclosure of mental health difficulties to one’s social circle could lead to fear of one’s belonging to this group being threatened, whereas disclosing to someone outside of one’s social circle does not threaten one’s belonging to the social group. Along these lines, a desire for confidentiality (i.e., a desire to prevent disclosure of the stigmatizing information to one’s social circle) could be an important predictor of from whom someone is likely to seek help. 

Perceived confidentiality is an important factor in the decision to seek help for one’s mental health.^54,55^ People high in internalized stigma may be more likely to seek help from professional sources than people within their social network (i.e., family and friends or intimate partners), due to confidentiality concerns. Individuals with concealable stigmatized identities (such as mental illness) often show a strong desire to conceal the stigmatized identity.^51,56^ Individuals may be more likely to seek help from professional sources for personal/emotional problems due to increased confidence that what they say will be kept private due to patient/provider confidentiality and further removal from the individual’s own social circle.

While internalized stigma did not significantly predict willingness to seek help for personal emotional problems from professional sources, it did negatively predict willingness to seek help for suicidal thinking from professional sources (as it did for the other targets). Again, the anticipated outcome of help-seeking may play an integral role in the differences in the relationship between internalized stigma and help-seeking for personal emotional problems and help-seeking for suicidal thinking. The potential consequences of disclosure of suicidal thinking (e.g., involuntary hospitalization) are much more extreme than the potential consequences of disclosure of a personal emotional problem. It is possible that, for personal emotional problems, the potential outcome of receiving mental health support may outweigh the possible negative outcome of negative perception from the provider. However, in the case of suicidal thinking, the potential negative outcomes (e.g., involuntary hospitalization) are more drastic. It is possible that the negative outcomes associated with disclosure of suicidal thinking could outweigh the potential positive outcome of receiving support.

Confidentiality could be even more important to people seeking help for suicidal thinking than for personal emotional problems, as many people experiencing suicidal ideation exhibit a strong desire to keep their ideation secret.^57^ Many people understand that patient-provider confidentiality can be broken in the case of suicidal ideation; this could lead individuals high in stigma to be concerned that self-disclosure of suicidal thoughts to professional sources could not be kept private, and word of their suicidal thinking could reach their social circles. Because of the high stigma attached to suicide,^58^ this fear of this potential negative outcome could drive the relationship between internalized stigma and help-seeking from professionals for suicidal thinking.

The finding that internalized stigma predicted lower likelihood of willingness to seek help for suicidal thinking across targets is sobering. Research indicates internalized stigma predicts increased suicidal behaviors.^57,59^ It seems that internalized stigma increases suicidal thinking while simultaneously leading to a lower likelihood of willingness to seek help for this suicidal thinking. This study’s findings contribute to the existing literature highlighting the importance of reducing internalized stigma in the interest of public health.

Lannin and colleagues^16^ found that self-stigma of seeking help negatively predicted help-seeking for psychological distress for both formal and informal targets. Conversely, the current study’s results indicate that internalized stigma does not negatively impact willingness to seek help for personal emotional problems from professional targets. This discrepancy highlights the fact that internalized stigma is a separate construct from self-stigma of seeking help, emphasizing that not all forms of stigma are equivalent and, thus, warrant study as unique constructs. It is not appropriate to universalize findings relative to one form of stigma related to psychological distress (e.g., self-stigma of seeking help) to others (e.g., internalized stigma of mental illness).

### Limitations

There are a number of limitations of this study that should be noted. First, the analyses relied on cross-sectional data. Consequently, the authors are not able to draw causal conclusions regarding the impact of internalized stigma on willingness to seek help. Next, the authors assessed beliefs around hypothetical help-seeking rather than actual help-seeking behaviors. While the measure of help-seeking utilized in this study is commonly used in the help-seeking literature, it remains possible that the reported likelihood of seeking help from different sources does not directly mirror actual behaviors. Finally, Sample 2 could be subject to sampling bias, as the participants in this sample had experience seeking help for their mental health before. It is possible that the relationships between internalized stigma and help-seeking could be different for individuals who had never sought help for their mental health. However, the authors mitigated this possibility by also recruiting from a sample of undergraduate students with and without a history of help-seeking (Sample 1), although over half of Sample 1 reported a history of help-seeking for mental health as well.

### Strengths

Despite the presence of several limitations, this study also featured multiple strengths that should be highlighted. First, replication of results across multiple samples of different demographic compositions lends the study’s findings credibility. Further, bifactor modeling allowed examination of the relationship between internalized stigma and willingness to seek help in a nuanced way, affording the ability to answer questions previously unexamined in the literature.

## Implications for Behavioral Health

The current findings add to extant research demonstrating that internalized stigma is an important predictor of willingness to seek help for one’s mental health. These results contribute new insight to the literature by demonstrating that the relationship between internalized stigma and willingness to seek help is not uniform across help-seeking targets and presenting problems. These findings have strong implications for behavioral health. The finding that internalized stigma did not predict lower likelihood of seeking help for a personal emotional problem from professionals but did negatively predict willingness to seek help from professionals for suicidal thinking indicates that not all individuals who seek help from professionals for their mental health are necessarily comfortable disclosing suicidal ideation. Therefore, behavioral health clinicians should not assume that someone will share their thoughts of suicide openly in session simply because they appear comfortable discussing other distressing topics. This highlights the importance of direct assessment of suicidal ideation. Likewise, the sole fact that someone has made the decision to seek help for their mental health does not mean that this person does not have internalized stigma that could negatively impact the therapeutic alliance, treatment progress, and likelihood of disclosure of suicidal thinking.^9,60,61^ Behavioral health clinicians should consider prioritizing the assessment of internalized stigma in their clients and addressing this as a treatment target.

### Future directions

Further research is warranted to better understand the findings of this study, as well as to elucidate possible implications for clinical practice. Future researchers should consider replicating this study in a sample of participants high in distress who have not previously sought help for their mental health. Conversely, it may be beneficial to extend this study by examining history of reported past help-seeking behaviors, rather than help-seeking attitudes/intention to seek help from each target. Additionally, further studies could replicate this study utilizing self-stigma of seeking help rather than internalized stigma of mental illness to further explore if the two constructs function uniquely in their relationships with willingness to seek help. Future research could shed light on whether the relationship between internalized stigma and willingness to seek help varies based on other presenting problems, such as more normalized mental health conditions like depression or anxiety vs highly stigmatized conditions such as psychosis.

As prior research has demonstrated that cultural and environmental factors have significant impacts on mental health help-seeking and stigma, future research should investigate how demographic variables (e.g., gender, race, bicultural identity) might impact the relationship between willingness to seek help and internalized stigma across help-seeking targets and presenting problems.^62–64^ Finally, various experiences associated with mental health treatment (such as voluntary vs involuntary treatment, quality of the therapeutic relationship, reactions of family and friends) could impact the relationship between stigma and willingness to seek help. Thus, further research could explore how the nature of past mental health treatment experiences may inform relationships between internalized stigma and willingness to seek help across presenting problems and help-seeking targets.

## Data Availability

The data that support the findings of this study are available on request to the corresponding author. . The data are not publicly available due to privacy or ethical restrictions. Study aims, predictions, and analytic plan were all pre-registered prior to conducting analyses and can be found at https://osf.io/mqpdb/overview?view_only=5db56bc7c1ec4daf8b28fad9a13a907c.
